# Mutations in *IDH1*, *IDH2*, and in the *TERT* promoter define clinically distinct subgroups of adult malignant gliomas

**DOI:** 10.18632/oncotarget.1765

**Published:** 2014-01-28

**Authors:** Patrick J. Killela, Christopher J. Pirozzi, Patrick Healy, Zachary J. Reitman, Eric Lipp, B. Ahmed Rasheed, Rui Yang, Bill H. Diplas, Zhaohui Wang, Paula K. Greer, Huishan Zhu, Catherine Y. Wang, Austin B. Carpenter, Henry Friedman, Allan H. Friedman, Stephen T. Keir, Jie He, Yiping He, Roger E. McLendon, James E. Herndon II, Hai Yan, Darell D. Bigner

**Affiliations:** ^1^ Department of Pathology, Duke University Medical Center, The Preston Robert Tisch Brain Tumor Center at Duke, and Pediatric Brain Tumor Foundation Institute at Duke, Durham, NC, USA; ^2^ Department of Biostatistics and Bioinformatics, Duke University Medical Center, Durham, NC, USA; ^3^ Department of Thoracic Surgery, Cancer Institute and Hospital Chinese Academy of Medical Sciences, Beijing, 100021, China

**Keywords:** TERT promoter, IDH1, IDH2, Glioma

## Abstract

Frequent mutations in *isocitrate dehydrogenase 1* and *2* (*IDH1* and *IDH2*) and the promoter *of telomerase reverse transcriptase* (*TERT*) represent two significant discoveries in glioma genomics. Understanding the degree to which these two mutations co-occur or occur exclusively of one another in glioma subtypes presents a unique opportunity to guide glioma classification and prognosis. We analyzed the relationship between overall survival (OS) and the presence of *IDH1/2* and *TERT* promoter mutations in a panel of 473 adult gliomas. We hypothesized and show that genetic signatures capable of distinguishing among several types of gliomas could be established providing clinically relevant information that can serve as an adjunct to histopathological diagnosis. We found that mutations in the *TERT* promoter occurred in 74.2% of glioblastomas (GBM), but occurred in a minority of Grade II-III astrocytomas (18.2%). In contrast, *IDH1/2* mutations were observed in 78.4% of Grade II-III astrocytomas, but were uncommon in primary GBM. In oligodendrogliomas, *TERT* promoter and *IDH1/2* mutations co-occurred in 79% of cases. Patients whose Grade III-IV gliomas exhibit *TERT* promoter mutations alone predominately have primary GBMs associated with poor median OS (11.5 months). Patients whose Grade III-IV gliomas exhibit *IDH1/2* mutations alone predominately have astrocytic morphologies and exhibit a median OS of 57 months while patients whose tumors exhibit both *TERT* promoter and *IDH1/2* mutations predominately exhibit oligodendroglial morphologies and exhibit median OS of 125 months. Analyzing gliomas based on their genetic signatures allows for the stratification of these patients into distinct cohorts, with unique prognosis and survival.

## INTRODUCTION

Gliomas are the most common primary malignant tumor of the central nervous system and account for 24% of brain tumors [1]. Tumor grades range from Grade I to Grade IV and are based on histopathological and clinical criteria established by the World Health Organization (WHO) [1, 2]. Grade I tumors are relatively benign and are circumscribed tumors that display a favorable prognosis with 94% of patients surviving at 5 years and 91% at 10 years [1]. Grade II gliomas are diffusely infiltrative and can be divided into astrocytomas and oligodendrogliomas. These tumors have the inherent ability to progress to higher grade gliomas. In addition to Grade II and III astrocytomas and oligodendrogliomas, another subtype of glioma presents with a histological appearance of both oligodendrogliomas and astrocytomas. These “mixed histology” tumors, or oligoastrocytomas also have the ability to progress from Grade II to Grade III tumors. The Grade III astrocytomas have the ability to further progress into secondary Grade IV glioblastomas (GBM), which exhibit a poorer prognosis than the grade III astrocytomas. As first described by Scherer in 1940 [3] secondary GBM arises as a progression from Grade II and Grade III tumors, whereas primary GBM arises *de novo* and has a dismal median OS of 15 months [1]. The progression between grades along with the potential for mixed histology presents neuropathologists with diagnostic challenges that often rely on subjective measures. Consequently, diagnoses among different pathologists and institutions have weak correlations that may result in variable treatment and management of each tumor grade [2, 4]. The subjective nature of these analyses stresses the importance of an accurate, unbiased, and objective means of diagnosis. This is crucial for stratification of patients with biologically similar tumors in clinical trials, and could aid in the selection of targeted therapeutic regimens. The discovery of biomarkers that objectively identify each tumor's unique molecular signature is a necessary next-step in managing patient outcomes more effectively. Genetic signatures performed on pathologically relevant tissues will be a potentially useful supplement to clinicians in refining and clarifying patient stratification.

Characterization of the genetic landscape of gliomas has been at the forefront of cancer research in order to better aid prognostication and classification of clinical outcomes [5, 6]. High-throughput screens have paid particular attention to understanding the genomic variability between each subgroup of glioma. The Cancer Genome Atlas and other groups, including ours, have begun to identify the molecular subgroups of these tumors and delineate which tumor types harbor which mutations [5-12]. For example, *IDH1/2* mutations that occur frequently in secondary GBMs (>50%) are infrequent in primary GBMs (<5%) [8, 12].

Recent findings have established frequent mutations in the promoter region of *telomerase reverse transcriptase* (*TERT*) in a multitude of cancers, including melanomas, liposarcomas, bladder cancer, urinary tract cancers, and gliomas [13-19]. *TERT* is a subunit of the telomerase enzyme that, when expressed, allows cells to avoid senescence. This is especially noted as *TERT* is mutated in high frequencies in cells with low rates of self-renewal, such as melanocytes, urothelial cells, and glial cells [14-16, 20, 21]. Of interest to glioma genomics, *TERT* promoter mutations occur in 70-80% of primary GBMs and >70% of oligodendrogliomas, but occur less frequently in both lower grade astrocytomas and most oligoastrocytomas [16, 17, 22].

The discovery of *TERT* promoter mutations in these subsets of gliomas creates an opportunity for genomics to supplement histopathological analysis, especially when combined with *IDH1/2* mutation status. Here, we have assessed the characteristic variance between *IDH1/2* and *TERT* promoter mutations among several glioma subtypes that help refine the diagnosis of gliomas. The assay, based upon three polymerase chain reactions (PCR), provides pathologists with a manageable and reliable diagnostic supplement in the form of a simple, yet robust genetic signature unique to each tumor type.

## RESULTS

### TERT promoter mutations are frequent in primary GBMs and oligodendrogliomas but uncommon in lower grade astrocytoma.

**Fig 1:**
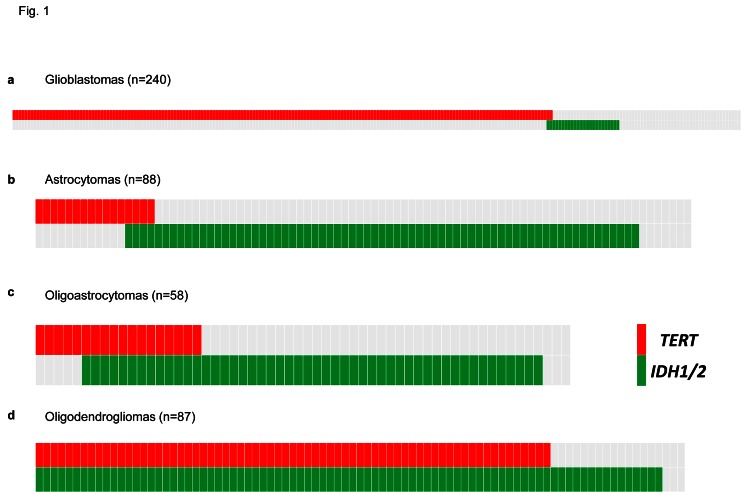
Distribution of *TERT* promoter and *IDH1/2* mutations in a panel of 473 adult gliomas Mutational analysis of 473 adult gliomas for *TERT* promoter and *IDH1/2* mutations. Data are from 240 Grade IV GBM (A), 88 Grade II-III astrocytomas (B), 58 Grade II-III oligoastrocytomas (C), and, 87 Grade II-III oligodendrogliomas (D). Mutation status is indicated by color shading, with gray coloring indicating wild type sequence, red indicating mutations in the *TERT* promoter, and green indicating mutations in *IDH1/2*.

To assess the prevalence and prognostic impact of *TERT* promoter mutations we sequenced the proximal *TERT* promoter hotspot mutations (C228T and C250T) in 473 adult gliomas. We identified *TERT* promoter mutations in 281 (59.4%) tumors (Fig. 1). In agreement with previous studies [16, 18, 23], we identified *TERT* promoter mutations in 74.2% of grade IV GBMs (178/240). *TERT* promoter mutations were also common in oligodendrogliomas (79.3%); however, *TERT* promoter mutations were less frequently identified in Grade II-III astrocytomas (18.2%, 16/88). Furthermore, we observed a moderate frequency of *TERT* promoter mutations in oligoastrocytomas (31.0%, 18/58). As expected, GBMs were diagnosed in older patients when compared to other histologic subtypes studied here (Table 1). Within each tumor type, *TERT* promoter mutations were associated with an older age at diagnosis (Table 2).

**Table 1:** Clinical Characteristics of Cohort

	GBM (N=240)		A (N=88)		OA (N=58)		O (N=87)	
**Age (mean, SD)**	54.9	13.4	39.0	10.4	42.0	13.2	41.2	11.2
Gender Male Female								
	146	60.8%	54	61.4%	36	62.1%	60	69.0%
	94	39.1%	34	38.6%	22	37.9%	37	31.0%
**Grade** II III IV								
	0	0.0%	40	45.4%	28	48.3%	44	50.6%
	0	0.0%	40	54.6%	30	51.7%	43	49.4%
	240	100.0%	0	0.0%	0	0.0%	0	0.0%
**Diagnosis Status** Newly Diagnosed Recurrent Not Available								
	132	55.0%	50	56.8%	30	51.7%	42	48.3%
	65	27.1%	35	39.8%	24	41.4%	31	35.6%
	43	17.9%	3	3.4%	4	6.9%	14	16.1%
***TERT* Status** Mutant Wildtype								
	178	74.2%	16	18.2%	18	31.0%	69	79.3%
	62	25.8%	72	81.9%	40	69.0%	18	20.7%
***IDH1/2* Status** Mutant Wildtype								
	24	10.0%	69	78.4%	50	86.2%	84	96.5%
	216	90.0%	19	21.6%	8	13.8%	3	3.5%
***TERT*-*IDH1/2* Status** *TERT* wt / *IDH* wt *TERT* wt / *IDH* mut *TERT* mut / *IDH* wt *TERT* mut / *IDH* mut								
	40	16.7%	7	7.9%	3	5.2%	3	3.5%
	22	9.2%	65	73.9%	37	63.8%	15	17.2%
	176	73.3%	12	13.6%	5	8.6%	0	0.0%
	2	0.8%	4	4.6%	13	22.4%	69	79.3%
**1p/19q Status** Wildtype/Wildtype Wildtype/19q 1p/Wildtype 1p/19q Not Available								
	0	0.0%	21	23.9%	26	44.8%	9	10.4%
	0	0.0%	7	7.9%	11	19.0%	0	0.0%
	0	0.0%	0	0.0%	2	3.5%	0	0.0%
	0	0.0%	2	2.3%	12	20.7%	47	54.0%
	240	100.0%	58	65.9%	7	12.1%	31	33.6%

**Table 2:** Age at diagnosis in gliomas as determined by *TERT* promoter genotype

		*TERT* Mutant	*TERT* WT	p-value
**GBM**	Age (mean ± SD) median yrs	57 ± 12	49 ± 16	0.0003
		57	52	
**A**	Age (mean ± SD) median yrs	44 ± 10	38 ± 10	0.0379
		43	36	
**OA**	Age (mean ± SD) median yrs	50 ± 12	38 ± 12	0.0008
		50	35	
**O**	Age (mean ± SD) median yrs	42 ± 10	37 ± 14	0.0933
		40	33	

### Co-occurring mutations in TERT promoter and IDH1/2

*IDH1/2* mutations are a well-established molecular feature of gliomas [12]. To define the co-occurrence of *IDH1/2* mutations and the presence of *TERT* promoter mutations, we determined the status of *IDH1* and *IDH2* mutations in the same cohort of 473 gliomas and identified mutations in 47.9% (227/473) of tumors (Fig. 1 and Table 1). *IDH1/2* mutations were much less prevalent among GBMs (10%), and much more common in Grade II-III astrocytomas (78.4%), oligoastrocytomas (86.2%) and oligodendrogliomas (96.5%). *TERT* mutations occurred in the absence of *IDH1/2* mutations in GBMs (73.3%, 176/240). However, in oligodendrogliomas, the *TERT* promoter mutation always occurred in the setting of the *IDH1/2* mutation, which is frequent in both oligodendrogliomas and astrocytomas (Fig. 1) [12]. The cross-tabulation of *TERT* promoter and *IDH1/2* mutations aligned with three of the four histologic subtypes. GBMs were characterized as primarily *TERT* promoter mutant/*IDH* wildtype (73.3%), Grade II-III astrocytomas were predominantly *TERT* promoter wildtype/*IDH* mutant (73.9%), and the majority of oligodendrogliomas mainly harbored mutations in both the *TERT* promoter and *IDH1/2* (79.3%). A majority of oligoastrocytomas (63.8%) exhibited the *IDH* mutation in the absence of *TERT* promoter mutations, much like Grade II-III astrocytomas; however, a fraction (22.4%) of oligoastrocytomas presented with both *TERT* promoter and *IDH1/2* mutations, similar to oligodendrogliomas (Fig. 1).

### TERT promoter and IDH1/2 mutations have distinct tumor distributions and are associated with OS

**Fig 2:**
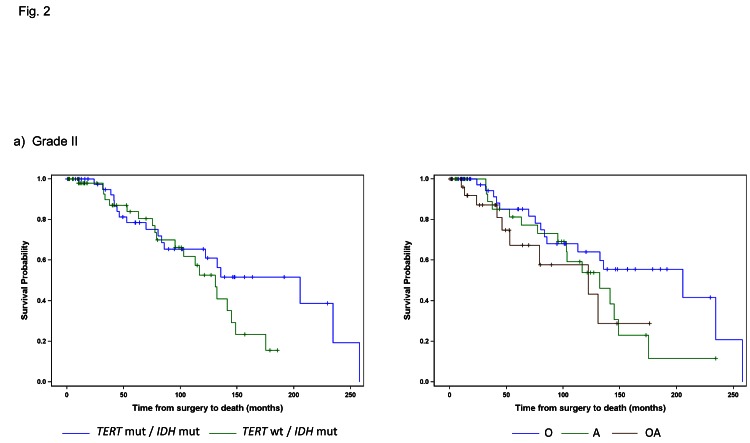

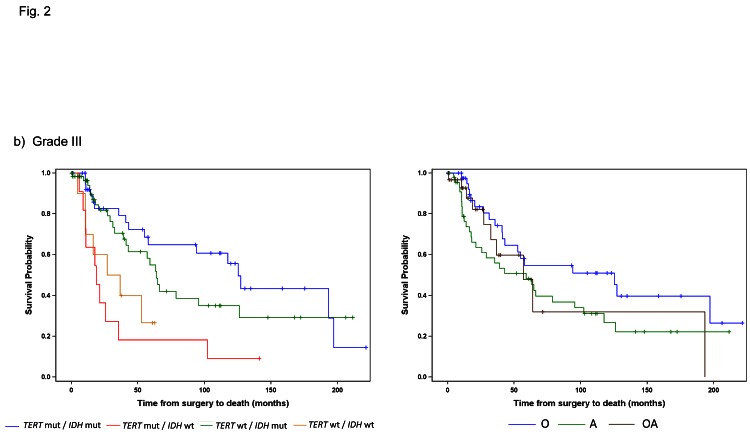

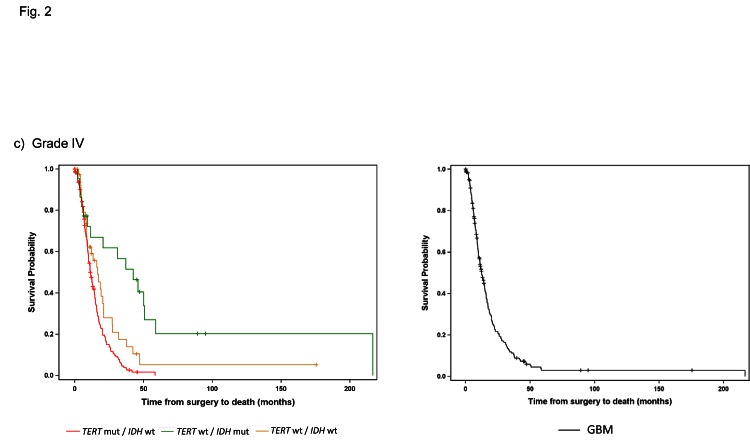
Overall Survival stratified by *TERT* promoter and *IDH1/2* mutational status and histology within each tumor grade Overall survival was represented by Kaplan Meier plots for individual WHO tumor grade: a) Grade II (n=103), b) Grade III (n=121), c) Grade IV (n=218). Only subgroups with at least 10 patients were included in the analyses. Tumors were represented by mutations status on the left (*TERT* promoter status / *IDH1/2* status) and histology on the right (A represents Astrocytomas, O represents Oligodendrogliomas, and OA represents Oligoastrocytomas).

We next sought to determine whether the combination of *TERT* promoter and *IDH1/2* mutations are associated with OS. Clinical information (survival, age at diagnosis, and histopathological diagnosis) was available for our cohort of 473 adult gliomas in both treated and untreated patients (Table 1). As grade is a well-known prognostic factor in glioma patients, we first investigated whether distinct tumor subgroups could be distinguished using only *TERT* promoter and *IDH1/2* mutation status within each grade (Fig. 2, Table 3). Among the 112 Grade II gliomas, 103 were characterized by either mutations in both *TERT* and *IDH* or *IDH* alone. The median OS of those tumors harboring mutations in both *TERT* promoter and *IDH1/2*, the predominant genetic signature in oligodendrogliomas, was longer than those tumors with an *IDH1/2* mutation only, typically seen in Grade II-III astrocytomas (206 months vs. 131 months), but this difference was not statistically significant (log-rank p=0.1754) (Fig. 2A). When stratified by histologic diagnosis, oligodendrogliomas had the best median OS among Grade II astrocytomas, oligodendrogliomas, and oligoastrocytomas, as expected (median OS 205 months).

**Table 3:** Summary of OS Stratified by *TERT* promoter and *IDH1/2* Mutational Status by Grade

	*TERT/IDH* status	Total	# failed	OS in months (95% CI)	1 year OS (95% CI)	2 year OS (95% CI)	5 year OS (95% CI)	10 year OS (95% CI)
**Grade II**								
	*TERT* WT / *IDH* MUT	57	20	130.7 (95.1, 145.0)	98% (86.4%, 99.7%)	98% (86.4%, 99.7%)	83.8% (67.3%, 92.5%)	52.7% (32.3%, 69.5%)
	*TERT* MUT / *IDH* MUT	46	18	205.5 (85.8, 257.9)	100%	97.4% (83.2%, 99.6%)	78.5% (61.5%, 88.6%)	65.4% (46.9%, 78.8%)
**Grade III**								
	*TERT* MUT / *IDH* WT	11	10	18.6 (8.7, 35.3)	63.6% (29.7%, 84.5%)	36.4% (11.2%, 62.7%)	18.2% (2.9%, 44.2%)	9.1% (0.5%, 33.3%)
	*TERT* WT / *IDH* WT	10	7	31.8 (4.4, -)	70.0% (32.9%, 89.2%)	60.0% (25.3%, 82.7%)	26.7% (4.8%, 56.3%)	NE*
	*TERT* WT / *IDH* MUT	60	24	63.8 (40.9, 126.2)	96.3% (85.8%, 99.1%)	81.9% (66.8%, 90.6%)	55.0% (37.3%, 69.6%)	35.1% (19.3%, 51.3%)
	*TERT* MUT / *IDH* MUT	40	16	127.3 (57.7, 197.2)	94.4% (79.6%, 98.6%)	84.9% (67.3%, 93.4%)	66.6% (46.5%, 80.6%)	57.3% (36.2%, 73.6%)
**Grade IV**								
	*TERT* MUT / *IDH* WT	176	158	11.3 (10.0, 13.1)	47.6% (39.8%, 54.9%)	15.0% (9.9%, 21.1%)	0.0%	0.0%
	*TERT* WT / *IDH* WT	40	30	16.6 (8.6, 21.0)	59.1% (41.5%, 71.1%)	27.9% (13.6%, 44.2%)	5.2% (0.5%, 19.5%)	5.2% (0.5%, 19.5%)
	*TERT* WT / *IDH* MUT	22	16	42.3 (9.1, 50.6)	67.0% (42.7%, 82.8%)	61.8% (37.7%, 78.9%)	20.3% (5.4%, 41.8%)	20.3% (5.4%, 41.8%)

* NE=Not Estimable

** Genotypes with frequencies less than 10 were not included

Among the 121 Grade III tumors, 60 (50%) had *IDH1/2* mutations alone and 40 (34%) had mutations in both the *TERT* promoter and *IDH1/2*. Those with mutations in both the *TERT* promoter and *IDH1/2* had the largest median OS (127 months), followed by those with an *IDH1/2* mutation only (median OS 64 months), and those with neither mutation (median OS 32 months). Tumors with mutations in the *TERT* promoter alone, which was the predominant signature present in primary GBMs had the poorest OS (median OS 19 months). Four distinct subgroups of Grade III gliomas were identified when stratified by the combination of the *TERT* promoter and *IDH1/2* mutation status (log-rank p=0.0008) (Fig. 2B). Oligodendrogliomas again had the best median OS when Grade III tumors were stratified by histologic subtypes (median OS 125 months), but OS did not significantly differ among the three histologic subtypes, which were astrocytomas, oligodendrogliomas, and oligoastrocytomas (log-rank p=0.1626).

A majority of the GBMs were characterized by mutations in the *TERT* promoter alone (73%), and this genetic signature also had the worst prognosis (median OS 11.3 months) (Fig. 2, Table 3). Those without mutation in either marker had only a slightly better outcome (median OS 17 months), while those with an *IDH1/2* mutation alone, the signature characteristic of Grade II-III astrocytomas and Grade IV secondary GBMs, had the best outcome among the Grade IV tumors (median OS 42 months). Within the primary and secondary GBMs, using the *TERT* promoter and *IDH1/2* alone, we were able to distinguish three significantly different subgroups (log-rank p<0.0001), and these associations remained when adjusting for the factors of age and diagnosis (Table 4). The *TERT* promoter mutation is associated with poorer OS in GBMs, and as shown in the multivariable model, this association was also evident among tumors without an *IDH1/2* mutation (HR: 1.9, 95% CI: 1.2-2.9).

**Table 4:** Cox Model Predicting Median Overall Survival in GBMs

	Parameter	DF	Hazard Ratio	95% Lower Confidence Limit
*TERT*	Mutant vs. Wildtype	1	1.901	1.244
*IDH1*	Mutant vs. Wildtype	1	0.496	0.251
Tumor Status	Newly Diagnosed vs. Recurrent	1	0.481	0.340
Age		1	1.019	1.004

* 198 tumors with all covariates available are included in the model

**Fig 3:**
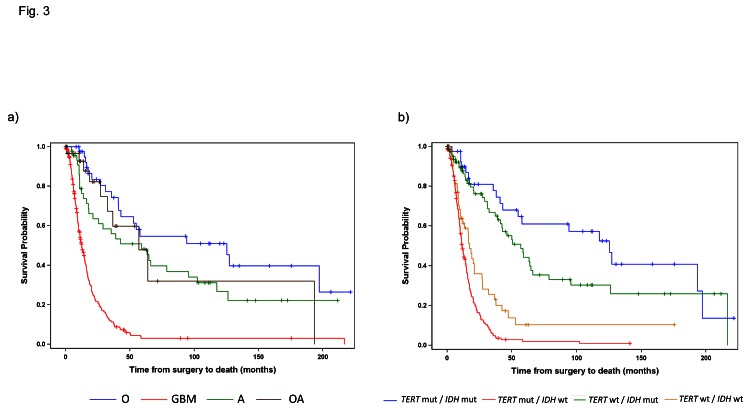
Overall Survival stratified by *TERT* promoter and *IDH1/2* mutational status and histology among Grade III and IV patients Overall survival was represented by Kaplan Meier plots stratified by a) histology (A represents Astrocytomas, O represents Oligodendrogliomas, OA represents Oligoastrocytomas, and GBM represents Glioblastoma) and b) *TERT* promoter / *IDH1/2* mutation status for all Grade III and Grade IV gliomas analyzed in this study.

Given that both Grade III and Grade IV gliomas were successfully stratified into distinct subgroups based on *TERT* promoter and *IDH1/2* mutational status, and that each signature was associated with a similar median OS within grade, the effect of histology and genetic signature on OS was also examined across the Grade III and IV gliomas together (Fig. 3, Table 5A). When Grade III and IV gliomas were examined based on histology, GBMs predictably had by far the worst prognosis, and oligodendrogliomas experienced the best survival outcome; however, OS among the Grade III astrocytomas and oligoastrocytomas was similar and difficult to distinguish (Fig. 3A and Table 5A). Nevertheless, when genetic signatures were applied to the same cohort of tumors, four distinct clinical subgroups emerged (Fig 3B). As observed, within Grade III and IV gliomas separately, tumors with mutations in both *TERT* and *IDH1/2* had the best median OS (oligodendroglioma signature), followed by those with an *IDH1/2* mutation only (Grade II-III astrocytoma and secondary GBM signature). Both tumors without mutation in either marker and those tumors with a *TERT* promoter mutation alone had a poorer prognosis, with the latter signature having the worst median OS. The strength of the association between OS and *TERT*/*IDH1/2* mutational status is similar to that of OS and histology (Generalized R^2^: 0.3132 and 0.2704, respectively).

**Table 5A:** Summary of OS Stratified by Histology in Grades III and IV

Histology	Total	# failed	OS in months (95% CI)	1 year OS (95% CI)	2 year OS (95% CI)	5 year OS (95% CI)	10 year OS (95% CI)
GBM	240	206	12.4 (10.9, 14.6)	51.2% (44.5%, 57.5%)	21.7% (16.4%, 27.6%)	3.0% (1.1%, 6.6%)	3.0% (1.1%, 6.6%)
A	48	29	59.0 (18.0, 95.6)	78.8% (63.2%, 88.4%)	63.5% (46.9%, 76.2%)	48.2% (32.1%, 62.5%)	26.7% (13.2%, 42.3%)
OA	30	9	56.9 (27.1, 193.3)	96.6% (77.9%, 99.5%)	85.8% (61.1%, 95.3%)	49.9% (19.4%, 74.4%)	33.3% (6.6%, 64.1%)
O	43	19	125.2 (40.9, -)	97.5% (83.5%, 99.6%)	83.5% (66.8%, 92.3%)	54.7% (36.1%, 69.9%)	51.0% (32.6%, 66.8%)

## DISCUSSION

Our analysis of this tumor cohort expands upon previous reports identifying frequent *TERT* promoter mutations in gliomas [16-18, 22, 23], examines the association between *TERT* promoter and *IDH1/2* mutations in glioma, and assesses their joint influence on OS. Utilizing a combined analysis of *IDH1/2* and *TERT* promoter mutations in adult glioma, we have derived a greatly expedited and simplified genetic signature of three common glioma subtypes, namely Grade II-III astrocytomas, oligodendrogliomas, and GBMs. Additionally, we show that oligoastrocytomas can be further classified.

Among patients with GBMs, we showed that the largest fraction of GBMs present with *TERT* promoter mutations. *IDH1/2* mutations are infrequent in these tumors and cluster within secondary GBMs. Three distinct subgroups were defined by the presence or absence of *TERT* promoter and *IDH1/2* mutations. Where patients harboring tumors with *TERT* promoter mutations alone had the poorest OS (median 11.3 months), patients with tumors bearing no mutations in either *TERT* or *IDH1/2* had a slightly better survival (median 16.6 months), and GBMs with *IDH1/2* mutation alone resulted in the best survival (median 42.3 months). Furthermore, these associations remained after adjustment for factors such as age. *TERT* promoter mutations predicted poorer OS outcome in a multivariate model even in GBMs without *IDH1/2* mutations. This finding is in contrast with previous studies that did not report a significant difference in OS between *TERT* promoter-mutated and *TERT* promoter-wildtype non *IDH* mutated GBMs [23]. This finding will be of particular interest to clinicians as it may provide a tool to stratify non *IDH1/2* mutant GBMs and suggests that combined *IDH1/2* and *TERT* promoter genotyping will be useful for patient management. Because of variable treatment among these histological brain tumor groups, further analyses must include large cohorts of standardized treatment arms and measurements of other genetic features such as *MGMT* status, *EGFR* wildtype amplification, and the presence of *EGFRvIII* to confirm the validity of our findings. At a minimum, our current findings warrant further investigation and confirmation by other investigators. Also, genetic alterations of the *TERT* promoter may be particularly relevant given the development of therapeutics targeted against telomerase. Telomerase inhibitors have shown promise for treating GBM in preclinical models and are currently under investigation in clinical trials for several types of cancer [24-27].

Conversely, *IDH1/2* mutations in Grade II-III astrocytomas are frequent while *TERT* promoter mutations are uncommon. Grade II-III oligodendrogliomas have frequent co-occurring mutations in the *TERT* promoter and *IDH1/2*. We provide evidence that over 86% of oligoastrocytomas in this cohort contain genetic signatures representative of either astrocytoma (*IDH1/2* mutations alone*)* or oligodendroglioma (*TERT* promoter*/IDH1/2*), signatures that we show are associated with OS. Reproducibility of oligoastrocytoma diagnosis by histology alone displays variable diagnoses between neuropathologists within and among different institutions [2, 4, 28]. The presence of the TERT promoter and IDH1/2 mutational status may be particularly useful to refine the classification of “mixed” oligoastrocytomas.

In addition to demonstrating the robust nature of these mutational patterns, we have further established that these genetic signatures are reliable when compared to the OS of patients derived from conventional histopathological diagnosis. As shown in Figures 2 and 3, mutations in the *TERT* promoter and *IDH1/2* effectively stratify patients into reproducible subgroups based on survival. This phenomenon was independent of grade among high grade astrocytomas as Grade III and Grade IV tumors mimicked this relationship when analyzed independently (Fig 2). Furthermore, the strength of these genetic signatures and their association with OS is illustrated by a slightly higher R^2^ (0.3132 vs. 0.2704) than by histology alone.

Two clinical subgroups exist among Grade II tumors in the current cohort, as the power of the survival analysis was limited due to the smaller number of low grade gliomas. The Grade II tumors exhibited genetic signatures with mutations in *IDH1/2* alone, and tumors with mutations in the *TERT* promoter and *IDH1/2.* Both subgroups had a more favorable prognosis, with a median OS of 130.7 months in tumors with *IDH1/2* mutations alone, and median OS of 205.5 months among patients whose tumors harbored *TERT* promoter and *IDH1/2* mutations. No Grade II tumors exhibited *TERT* promoter mutations alone.

Within Grade III-IV gliomas, those patients with the *TERT* promoter mutations alone had the poorest prognosis (median 11.5 months), while tumors bearing the events typically representative of astrocytomas (*IDH1/2* mutation) had a more favorable prognosis (median 56.9 months). Tumors harboring mutations typically seen in oligodendroglioma (both *TERT* promoter and *IDH1/2* mutation) had a more favorable prognosis (median 125.2 months). Tumors that did not harbor mutations in either the *TERT* promoter or *IDH1/2* comprised a unique clinical group with a short OS (median OS 17.2 months) that was distinct from *TERT* promoter mutated gliomas (median OS 11.5 months) (Table 5B). As these gliomas, wildtype for both *TERT* promoter and *IDH1/2* mutations, represented a clinically distinct unit (Fig. 2B and 2C) further investigation is required to delineate critical driver mutations in this subset of gliomas.

**Table 5B:** Summary of OS Stratified by *TERT* promoter and *IDH1/2* Mutational Status in Grades III and IV

			OS in months (95% CI)	1 year OS (95% CI)	2 year OS (95% CI)	5 year OS (95% CI)	10 year OS (95% CI)
*TERT* MUT / *IDH* WT	187	168	11.5 (10.0, 14.0)	48.5% (41.0%, 55.7%)	16.4% (11.3%, 22.4%)	1.9% (0.5%, 5.5%)	1.0% (0.1%, 4.4%)
*TERT* WT / *IDH* WT	50	37	17.2 (10.5, 26.2)	61.4% (45.9%, 73.7%)	35.9% (21.8%, 50.2%)	10.4% (3.0%, 23.2%)	10.4% (3.0%, 23.2%)
*TERT* WT / *IDH* MUT	82	40	56.9 (38.8, 66.2)	87.8% (77.8%, 93.5%)	76.2% (63.9%, 84.8%)	44.3% (30.6%, 57.1%)	30.3% (18.0%, 43.6%)
*TERT* MUT / *IDH* MUT	42	18	125.2 (54.7, 197.2)	92.2% (77.8%, 97.4%)	83.2% (66.2%, 92.1%)	62.7% (43.4%, 77.1%)	53.9% (33.9%, 70.2%)

It is of interest to note that within each tumor type, a minority of tumors bore the genetic signature typically associated with other histological subtypes. In particular, 13.6% (12/88) of Grade II-III astrocytomas bore *TERT* promoter mutations alone and occasional Grade II-III astrocytomas harbored both *TERT* promoter and *IDH1/2* mutations (4/88, 4.6%). This suggests that at least genetically, these tumors may be more similar to GBM and oligodendroglioma, respectively. Oligodendrogliomas were almost exclusively *TERT* promoter and *IDH1/2* mutated (79.3%, 69/87), but a fraction, 17.2% (15/87) harbored mutations in *IDH1/2* alone. In our cohort, no oligodendroglioma cases harbored *TERT* promoter mutations alone. A minor fraction of GBMs (0.8%, 2/240) contained mutations in both the *TERT* promoter and *IDH1/2* suggesting they were treated oligodendrogliomas that were diagnosed as small cell GBMs.

Loss of chromosomal arms 1p and 19q is a well-known genetic event associated with oligodendrogliomas that many neuropathologists use as a reliable test for diagnosing oligodendroglioma, a tumor generally associated with favorable prognosis and response to chemotherapy [29-32]. As a secondary analysis, the 69 oligodendrogliomas with 1p/19q status available were analyzed for an association with *TERT* promoter/*IDH1/2* mutational status. All 44 oligodendrogliomas with *TERT* promoter and *IDH1/2* mutations also had the 1p/19q allelic deletions and all but 3 of the 47 tumors with 1p/19q allelic losses also contained both *TERT* promoter and *IDH1/2* mutations, indicating that *IDH1/2* and *TERT* promoter mutational analysis may be a comparable prognostic markers to 1p and 19q in oligodendrogliomas (Fisher exact p<0.0001).

This study supports genotyping of *TERT* promoter and *IDH1/2* in gliomas as a rapid economical test requiring little tumoral DNA that could help inform clinicians as to the predicted OS of these tumors that may differ from their predicted outcomes based on conventional histology alone. The *TERT* promoter mutations analyzed in this study lay only 22 base pairs apart, allowing for PCR amplification in a single amplicon. Additionally, the most frequent mutations in *IDH1* and *IDH2* occur in hotspot residues located at resides R132 and R172, respectively. Combined together, these three PCR amplicons allow for expedient turnaround, objective interpretation, and vast economic advantages to glioma patients.

The *TERT* promoter*/IDH1/2* mutational profiles of each tumor type can be used in several aspects of the clinical process including stratification of patients, examination of therapeutic response, and selection of treatment, among others. Given the background genes previously discovered in glioma, we hypothesize *TERT* promoter and *IDH1/2* mutations as the major driver genes that are consistently found in low-grade and high-grade adult gliomas. These gene mutation assays will support and expedite the diagnosis of brain tumors while supplementing histopathological evaluation. Measurement of these biomarkers could further increase the fidelity of glioma diagnosis in a rapid and cost-effective manner. Furthermore, the simplicity and affordability of these tests underscore their importance as a tool to aid neuropathologists in glioma diagnosis. Notably, these signatures can be applied to cases that present atypical morphologic features in standard histopathological analysis. Taken together these findings simplify the genetic classification of glioma. The ability of these genetic signatures to stratify patients will refine and clarify the diagnostic accuracy of pathologists by supplementing standard histopathological criteria with genetic mutational analysis.

## METHODS

### Sample Collection, Processing, and Sequencing

Adult glioma (18 ≥ years old) and corresponding clinical information were obtained with consent and Institutional Review Board approval from the Preston Robert Tisch Brain Tumor Center BioRepository at Duke University in accordance with the Health Insurance Portability and Accountability Act. Newly diagnosed versus recurrent glioma status and vital status were determined by clinical chart review. Fresh frozen tissue sections (first and last sections from the block, stained with hematoxyline and eosin) were reviewed by a board-certified neuropathologist (REM) to confirm original clinical histopathologic diagnosis and to ensure intervening studied sections contain ≥ 80% tumor cells. DNA was extracted from 240 Grade IV GBMs, 88 Grade II and Grade III astrocytomas, 58 Grade II and Grade III oligoastrocytomas, and 87 Grade II and Grade III oligodendrogliomas. Of the 473 tumors, 160 gliomas had been analyzed in our previous studies of the *TERT* promoter [16]. Isolated DNAs were PCR amplified for the *TERT* promoter, exon 4 of *IDH1*, and analyzed via Sanger sequencing for 473 tumors as described previously [12, 16, 33]. Additionally, on those cases that did not harbor mutations in *IDH1* we amplified exon 4 of *IDH2* and analyzed them via Sanger sequencing. 1p and 19q copy number was evaluated by microsatellite marker analysis and via 1p and 19q FISH testing in a certified clinical laboratory as described previously [12, 29, 34].

### Statistical Methods

Clinical and demographic characteristics at the time of diagnosis were summarized for all patients and stratified by histologic tumor type. Means and standard deviations were used to describe interval variables, whereas frequency distributions were used to describe categorical variables. Unpaired t-tests were used to compare the mean age of patients with and without *TERT* promoter mutations. The Kaplan-Meier estimator was used to describe OS. OS was defined from time of surgery to death or last follow-up. Multivariable Cox models were used to assess the effect of *TERT* promoter and *IDH1/2* mutations on OS adjusting for baseline tumor characteristics. The generalized R^2^ statistic was used to assess the strength of association between covariates. Associations between categorical variables were analyzed using Fisher exact tests.
